# Reopening Schools during the COVID-19 Pandemic: Overview and Rapid Systematic Review of Guidelines and Recommendations on Preventive Measures and the Management of Cases

**DOI:** 10.3390/ijerph17238839

**Published:** 2020-11-27

**Authors:** Giuseppina Lo Moro, Tiziana Sinigaglia, Fabrizio Bert, Armando Savatteri, Maria Rosaria Gualano, Roberta Siliquini

**Affiliations:** 1 Department of Public Health Sciences and Paediatrics, University of Torino, 10126 Torino, Italy; giuseppina.lomoro@unito.it (G.L.M.); tiziana.sinigaglia@unito.it (T.S.); armando.savatteri@unito.it (A.S.); mariarosaria.gualano@unito.it (M.R.G.); roberta.siliquini@unito.it (R.S.); 2 AOU City of Health and Science of Turin, 10126 Torino, Italy

**Keywords:** COVID-19, schools, reopening, prevention and control

## Abstract

Given the limited evidence of school closure effectiveness in containing the pandemic and the consequences for young people, reopening schools with appropriate measures is essential. This overview aimed to describe the main measures planned for the 2020–2021 academic year within the WHO European Region. A rapid systematic review of scientific databases was also performed. The websites of the government, Ministry of Health, and Ministry of Education of European Region countries were searched through 1 October for official documents about the prevention and management of suspected cases/confirmed cases in primary and secondary schools. To find further suggestions, a rapid systematic review was conducted through 20 October searching Pubmed, Scopus, and Embase. There were 23 official documents. France, Luxembourg, Malta, Ireland, Italy, Portugal, the UK, Spain, and San Marino were considered. Performing the rapid review, 855 records were identified and 7 papers were finally selected. The recommendations mostly agreed. However, there was no consensus on the criteria for the return to school of students that tested positive, and the flexibility between attendance at school and remote education for high-risk children often varied. School closure was commonly considered as the very last resort for COVID-19 control. Studies are required to evaluate the impact of different recommendations during this autumn term.

## 1. Introduction

Up to early October 2020, coronavirus disease 19 (COVID-19) presented a cumulative total of over 34 million cases and over 1 million deaths worldwide. The majority of deaths have been reported in the World Health Organization (WHO) Region of the Americas (55%) and the WHO Region of Europe (23%). In numerous countries, especially in the European Region, the second wave has been greater than the past peaks, partially due to an improvement in surveillance efficiency. Focusing on the European Region, the incidence of new cases is constantly increasing, with France, the Russian Federation, the United Kingdom (UK), Spain, and Israel reporting the greatest number of new cases in the first week of October [1].

In this context, less than 5% of cases in the European Economic Area and the UK have been reported to occur in young people aged 18 years and under [2]. Specifically, children often present an asymptomatic infection, during which it is not well known how infectious children are. Instead, symptomatic children can spread the infection similarly to adults [2]. Evidence indicates that no age correlation with viral load exists, thus suggesting that children and young people can carry the same high levels of virus [3].

Although outbreaks in schools have been reported, their detection is extremely difficult because of the poor presentation of symptoms among younger people [2], and there is little evidence on the transmission dynamics in school settings [4]. The documented cases in schools indicated that child to child transmission in schools is not common and appropriate measures of prevention can potentially be effective in preventing transmission in the school setting [2]. Data about the effect of the school closure/reopening on the transmission in the community are conflicting and equivocal [2,5], and a wide range of impacts of school closures have been reported, from poor to substantial effects [5]. Recently, modelling studies have indicated that school closures alone would avoid only 2–4% of deaths, a percentage much smaller than other strategies of social distancing [5]. Such closures are not likely to be effective as a single control measure, but they should be matched with other physical distancing and public health response measures outside the school setting [2]. In the past months, some data about school reopening have shown that, despite the low-incidence period and hygiene measures promoted in schools, school outbreaks did occur [6,7]. However, the number of cases was lower compared to before school closures, thus indicating that containment measures in schools may be effective and should be implemented in the best possible way [6,8]. Interestingly, Sweden, which kept preschools and primary schools open, did not report greater numbers of hospitalized children due to COVID-19, although the overall epidemic has been reported to be severe [2]. However, indirect data suggest that children in Sweden were infected considerably more than similar countries, such as Finland [9].

Notably, strategies less disruptive than school closure must be considered in a context where restrictive distancing policies are enacted for long periods, as school closures can lead to very high costs both in economic and social fields [5]. In particular, school closures may have several consequences for children, adolescents, and their families, as schools are both an educational setting and a source of health and mental services, food assistance, obesity prevention, and support for maltreatment and homelessness [10]. For instance, the lockdown has resulted in several mental health issues in children and adolescents [11], and the school closure represented a lack of access to mental health services for young people in need [12]. Similarly, with schools being one of the most important sources of reported maltreatment, the school closure inhibited the reporting of child maltreatment [13].

Therefore, given the limited evidence of school closure effectiveness in containing the pandemic and the importance of the consequences of school closure on young people’s lives, it seems essential to implement preventive measures when reopening schools, along with clear strategies to manage potential cases and outbreaks in school settings, in order to reduce the transmission of COVID-19 and keep schools open.

In September, with the beginning of the academic year 2020/2021, the vast majority of the countries belonging to the WHO European Region fully reopened schools [14]. Thus, the aim was to describe and compare the main measures of the prevention and management of symptomatic or positive persons planned and implemented for the new academic year within the WHO European Region through an overview of official documentation released by the government institutions of countries belonging to the European Region and a rapid systematic review of guidelines and recommendations.

## 2. Materials and Methods

### 2.1. Official Documentation Released by Government Institutions

#### 2.1.1. Data Sources and Search Strategy

The present investigation was directed to all countries included in the European Region of WHO [15] in order to give a panoramic overview regarding reopening school guidelines and recommendations for the 2020/2021 academic year, specifically for primary and secondary schools. The authors searched the websites of the government, Ministry of Health, and Ministry of Education of each country of the European Region for official documents about the measures for the prevention and management of suspected cases/confirmed cases in school settings. The authors conducted the search between the 1 September and the 1 October 2020. No restriction on the publication date of the documents was applied.

The search strategy research was structured using the PICOS strategy, as can be seen in Table 1.

#### 2.1.2. Document Selection and Data Extraction

Documents were considered eligible if they reported official detailed guidelines or protocols on the reopening measures to be followed in school settings both to prevent the COVID-19 transmission and to manage suspected/confirmed cases of COVID-19 infection. The authors examined only documents written in English, French, Spanish, Italian, or Portuguese. Only state-level documents and documents referring to the academic year 2020/2021 (or autumn term) were included. Only documents on primary and secondary schools were considered eligible, while information about preschools, colleges, and universities were excluded. News, statements, and question and answer pages were excluded. Two authors (GLM and TS) independently screened the websites of the government, Ministry of Health, and Ministry of Education to identify relevant documents. Disagreements were resolved by consensus. Extracted data for preventives measures included publication date, information about attendance, masks, physical distancing, interactions and behaviors, hand hygiene, ventilation, cleaning and disinfection, physical activity, school transport, and canteens. Extracted data for management measures included publication date, information about the management of students (and staff) with symptoms, and information about the management of students (and staff) who test positive.

### 2.2. Rapid Systematic Review in Scientific Databases

#### 2.2.1. Data Sources and Search Strategy

In order to find further suggestions for preventive and management measures, a rapid systematic review [16,17] was conducted following the Preferred Reporting Items for Systematic Reviews and Meta-Analyses (PRISMA) checklist by searching the Pubmed, Scopus, and Embase databases for studies that presented guidelines, recommendations, or protocols for the reopening of primary and secondary school during the COVID-19 pandemic. Studies published in 2020 up to 20 October were included. Search terms and keywords were matched with medical subject heading (MeSH) terms. Search terms for three different themes were linked with AND: COVID-19 AND schools AND reopening. A full list of search terms and keywords is described in the Supplementary Material. Duplicates were removed.

#### 2.2.2. Document Selection and Data Extraction

Studies were considered eligible if they reported guidelines, recommendations, or protocols for the reopening of primary and secondary schools. Differently from the search for official documents described above, papers referring to the reopening of schools before the new academic year 2020/2021 were included. Only papers that reported measures concerning the prevention of COVID-19 transmission or the management of suspected/confirmed cases were considered eligible. The authors examined only studies written in English, French, Spanish, Italian, or Portuguese. Commentaries and letters were excluded. Other exclusion criteria were studies on preschools, colleges, and universities and the unavailability of full texts. Authors chose the web application Rayyan of the Qatar Computing Research Institute (QCRI) as a tool for selecting and extracting relevant studies [18]. Three authors (GLM, TS, and AS) independently screened titles and abstracts to identify relevant studies, and three authors (GLM, AS, and FB) independently applied the inclusion and exclusion criteria to the full texts. Disagreements were resolved by consensus and reasons for exclusion were documented. Extracted data correspond with the official documentation’s categories.

## 3. Results

Overall, due to language restrictions, the countries included in this overview were the following: France, Ireland, Italy, Luxembourg, Malta, Portugal, Spain, the United Kingdom, and San Marino. Table 2 provides essential and relevant information for these countries, including the date of the first lockdown measures, the number of COVID-19 cases, and the number of deaths up to school closure and school reopening for the academic year 2020/2021 [14,19,20,21].

The number of official documents identified and considered relevant in the present overview was 23. The documents belonged to the following countries: France (*n* = 2) [22,23], Luxembourg (*n* = 2) [24,25], Malta (*n* = 1) [26], Ireland (*n* = 4) [27,28,29,30], Italy (*n* = 5) [31,32,33,34,35], Portugal (*n* = 3) [36,37,38], the UK (*n* = 2) [39,40], Spain (*n* = 3) [41,42,43], and San Marino (*n* = 1) [44]. These documents were published between May [26] and October [33], however the majority was published in August [22,26,27,28,30,31,34,42,44] and September [23,24,25,33,35,36,37,38,40,43]. All the documents were written in the official language of the country (documents of Luxembourg [24,25] were available in French and those of Malta [26] were available in English). Documents were accessed through the government websites in 4 cases [33,36,39,40], the Ministry of Health website in 4 cases [26,30,34,42], and the Ministry of Education website in 15 cases [22,23,24,25,27,28,29,31,32,35,37,38,41,43,44]. The level of detail was very heterogeneous between the documents. Overall, eight documents had information about the both prevention and management of cases [24,26,27,28,36,37,39,42], nine only about preventive measures [22,29,31,32,33,35,38,41,44], and six only about the management of suspected/confirmed cases [23,25,30,34,40,43].

**Figure 1:**
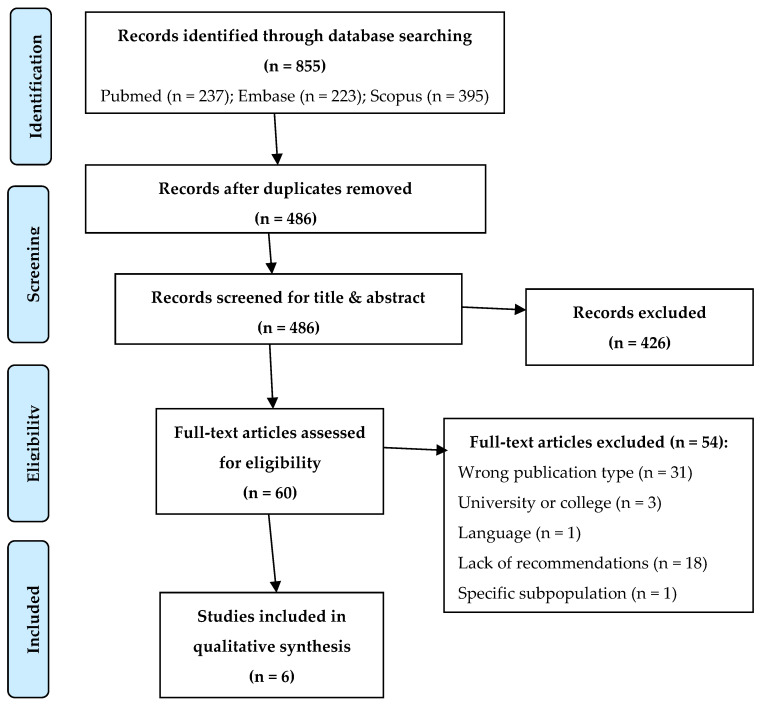
Rapid systematic review: selection process. From: Moher D, Liberati A, Tetzlaff J, Altman DG, The PRISMA Group (2009). Preferred Reporting Items for Systematic Reviews and Meta-Analyses: The PRISMA Statement. PLoS Med 6(7): e1000097, doi:10.1371/journal.pmed1000097.

Performing the rapid systematic review, a total of 855 records were identified. The study selection process is described in Figure 1. The selection resulted in seven studies included for the review. All the studies were available in English. The selected papers were published between June [45] and October [46]. Overall, the studies were from the UK [47], Germany [48], France [49], India [46], Norway [45], and the USA [50]. Three studies reported information about the both prevention and management of suspected/confirmed cases [45,46,48]; one study focused on preventive measures [41] and another on the management of cases [47]. Unlike institutional documents, most documents found by systematic research often contained fewer technical details.

### 3.1. Preventive Measures

#### 3.1.1. Official Documentation Released by Government Institutions

**Attendance and remote education:** All students are expected to return to school [22,39,41,42], however in some countries students at certified high risk for their medical conditions can use remote education [26,27,28,29,35,36,37], as well as high-risk teachers [24,26]. Some countries are more flexible, and schools can evaluate the opportunity to use hybrid education (both remote and in presence) and rotations—for instance, if available premises are not sufficient [24,26,31,32,41,42]. In other countries, remote education should be considered only in exceptional circumstances depending on the epidemiological situation [22,39]. In some documents, it is also clarified that students in quarantine should attend remote education [22,24,26,39].

**Masks:**Table 3 shows recommendations for the use of masks, both for students and staff. Overall, staff members (and other adults) should always wear a mask, especially if adequate distancing is impossible to achieve. Some exceptions are reported in Spain, where the mask is optional within stable coexistence groups [41,42], and the UK, where universal use is not recommended [39]. For students, the recommendations are usually different depending on age and situation: in primary schools, it is mostly not recommended or facultative, while in secondary schools it is usually mandatory, at least in common areas (Table 3). It is not recommended for students with medical or behavioral conditions incompatible with mask use [22,26,31,32,41,42].

**Physical distancing:** Physical distancing is considered one of the most important preventive measures in all documents. Distances range from a minimum of 1 m to a minimum of 2 m, and usually the distance that should be kept is higher between members of staff and students than between students (Table 3). All the reviewed documents suggest utilizing and reconfiguring all available spaces in order to maximize physical distancing, also signaling routes to follow, distances, and waiting points [31,36,37]. Class settings could be readapted, seating pupils side by side and facing forwards [37,39], removing unnecessary furniture from classrooms to make more space, and placing the least number of students possible in each classroom [26].

**Decrease interaction:** In all countries, school activities should be reorganized to limit contacts and avoid mixing between students and staff. Revise timetable considering staggering start/end times and use of different entrances around the school may decrease the crowding of students on arrival and departure [26,27,28,31,32]. Additionally, access to common areas should be carefully regulated—e.g., through staggered lunch times and breaks [24,26], fixed seats assignment [24], signaling the places to be occupied [36,37]. Wherever possible, students and staff must remain within the same classroom/cluster/group, whose composition should remain constant and keep separated as much as possible from other groups [24,27,28,29,37,39,41,42].

**Respiratory etiquette, interactions, and behavior:** All the countries provide instructions regarding respiratory etiquette, including sneezing/coughing in disposable tissues, disposed of immediately in a closed bin. If a tissue is not available sneeze/cough into flexed elbow, avoid physical contact (touching, hugging or shaking hands) and touching face and mask [32,36,37,41,42]. Other measures suggested are avoid touching publicly accessible objects (e.g., door handles or elevator buttons) with your full hand or fingers [26,37], encourage students to avoid behaviors that involve hand to mouth contact (putting pens/pencils in the mouth) [27,28,29], and discourage the sharing of personal and educational material [27,28].

**Hand hygiene:** Promotion and reinforcement of hand hygiene practice is strongly encouraged, and it is preferable to use warm water and soap in all countries. It is possible to use hand sanitizer but when hands are visibly dirt use water and soap [27,28,41,42], and dry with disposable paper or in air [22]. Hand hygiene should be rigorously monitored and performed at regular intervals, especially arrival at school, before eating or drinking, after using the toilet; after playing outdoors; when hands are physically dirty, after coughing/sneezing [27,28,36,37,39,41,42]. Hand sanitizer dispensers can be deployed more readily at exit and entry points of schools and classrooms [22,26,27,28,32] under the supervision of an adult in primary school or for students with special needs [22,26,39].

**Ventilation, cleaning and disinfection:** All the countries highlighted the importance of an adequate ventilation of all areas. For instance, ventilation should be ensured before entrance, during break, end of the day [22,41,42], at the beginning and ending of classes [24], during cleaning operation, and every 3 h [22]. Ventilation should last at least 15 min [22,41,42] and windows and/or doors should be kept open [24,26,41,42]. In the same way, cleaning and disinfecting are essential everywhere. Floors, surfaces, and all premises should be cleaned at least once per day [22,26,27,28], with additional cleaning on frequently touched surfaces (e.g., door handles) [22,24,27,28,32,37,39,41,42]. Toilets must be cleaned frequently [24,39], at least two [31,32] or three times a day [26,41,42]. Special attention should be given to the cleaning of premises and tools between groups/clusters [26,39,41,42]. In Malta, students in the same classroom and/or bubble should be encouraged to wipe down their desks and equipment before and after use [26].

**Physical activity at school:** Outdoor activities should be preferred [24,26,38,39,42], otherwise ventilation should be maximized and distancing among students should be kept [26,39], e.g., 2 [27,28,31,32] or 3 m [36,37]. In all countries, during physical activity, students should not wear masks or face coverings. Groups/clusters should be kept consistent during activities [26,39], individual sports should be preferred [31,32] and hand hygiene should be promoted [24,38]. Lastly, it is important to minimize equipment sharing and clean shared equipment [27,38].

**School transports:** Masks are mandatory [22,24,26,33,41,42,44], specifically above 11 years [22] or 6 years [24,33,41,42]. Additionally, students are advised to wear face coverings or masks in the UK (above 11 years) [39] and in Ireland (post primary students [28]. Staggered drop off/pick up times should be arranged [27,28,39], distancing should be maintained during boarding/disembarking and journey [26,33,36,39] and hand hygiene before boarding transport and again on disembarking ensured [39]. In Italy and Portugal, there is a maximum capacity of the vehicle of around two-thirds [33,36]. An adequate and frequent cleaning and ventilation of vehicles should be performed [26,33,36,39] and sanitizers in the vehicle should be available [26,33,36]. The use of seat markers is encouraged [33,36], and fixed seats for the entire school year can be assigned [41,42]. Retaining the same cohort/cluster of students on every journey is important [26,39]. Lastly, walking/cycling should be encouraged [27,28,41].

**School canteens:** School canteens are reopening in all countries. Among the main measures to be followed, it is clarified to wear a mask until sitting [24,36], keep distance [27,28,32,41,42], forbid buffet [24], stagger serving time [27,28,31,32,36,37], offer the chance to eat in classroom [24,31,36,41,42], organize separate meal time or areas for bubbles/clusters [27,28,41,42], and clean after every utilization [22,36,37,41,42].

#### 3.1.2. Rapid Systematic Review

**Attendance and remote education:** Remote education should be feasible in all educational facilities, as this might prevent potential exposure or transmission among staff and children. In the case of a high incidence of COVID-19 transmission divided classes, supplemented by online lessons should be implemented [48]. Alternatively, students could attend school on alternate weeks [47]. The guidelines of the French Pediatric society propose normal school attendance for children with chronic conditions, unless there is a validated exception by the child’s referring specialist [49]. Instead, Bonell et al. propose remote education for all children with underlying health conditions [47], whereas the Norwegian guidelines have provided a list of health conditions and comorbidities for which remaining at home is recommended [45].

**Masks:** Recommendations for the use of masks differ among documents (Table 4). Some authors propose mandatory masks at all times for both staff and students [46], for staff only [49] or when staff or students become ill and it is not possible to maintain a distance of two meters [45]. According to Simon et al., staff should wear a mask when distance rules cannot be observed, while students should always wear it if they are >10 years old. If a high incidence of COVID-19 cases occurs within the associated district, then students older than 6 years should wear masks [48].

**Physical distancing:** Although all the documents agree on the importance of physical distancing, different recommendations have been proposed, from at least 1 m [46] to 2 m [47] (Table 4). Means of increasing distancing include one-way systems in corridors and other shared spaces, zoning of playgrounds, using staggering, and dividing classes into subgroups [48]. Moreover, furniture within classrooms could be rearranged [46].

**Decrease interaction:** Schools should implement effective strategies to decrease interactions particularly among students, such as subgrouping/cohorting/bubbling classrooms [45,46,47,48]. Staff members should go to the classroom rather than children moving between rooms, so that the cohorts do not change [45,47]. Moreover, schools should make greater use of outdoor time and outdoor schooling [45].

**Respiratory etiquette, interactions, and behavior:** Scientific information regarding respiratory etiquette should be shared with staff/students/parents using all means, such as mails, telephonic calls, letters, pamphlets, etc. [46]. Norwegian guidelines recommend providing paper towels in readily accessible places for use by students/staff. If those are not accessible, pupils/staff should cough or sneeze into their elbow, avoid touching the face or eyes and washing their hands after close contacts. Shaking hands, hugging, and unnecessary physical contact must be avoided wherever possible [45].

**Hand hygiene:** Handwashing facilities should be easily accessible and widely distributed within the school premises and classrooms [46,47,48,49,50]. Education about their regular use should be encouraged [46], especially for kids under the age of 6 [49]. Johansen and colleagues give more detailed instructions regarding the procedure and when it should be performed (such as before leaving home and when they get home, when arriving at school/after-school program, after coughing/sneezing, after the use of a toilet, before/after meals, after outdoor activities/breaks, when hands are visibly dirty) [45]. Bonell and Ghate suggest the use of soap and sanitizer dispensers operated without touch or using elbow/foot [46,47]. Bonell, moreover, advises to use paper-towels dispensed using no/low-touch dispensers, as airflow-based hand-dryers can create virus-containing aerosols when used by infected individuals [47].

**Ventilation, cleaning, and disinfection:** Bonell, Ghate, and Simon highlight the importance of keeping airy and well ventilated all school premises opening doors and windows [46,47,48]. Simon et al. recommend hourly ventilation and, if necessary, the additional measurement of CO_2_ air content in the room and assessment air conditioning systems especially the proportion of fresh air or recirculated air [48]. Ghate and colleagues discourage the use of air conditioners [46]. Cleaning and disinfection practices should be increased especially for frequently hand-touched surfaces [45,46,47] and other frequently used objects such as toys, educational instruments, dining tables etc. [45]. Operators should use alcohol and chlorine-based disinfectants [45,47] and keep track of these procedures [46]. Simon et al. recommended to perform additional the daily disinfection of surfaces in the case of a high incidence (>50 new cases) of confirmed cases within the district [48].

**Physical activity at school:** Physical activity should be organized aiming to avoid close contact between students and individual activities should be preferred, maintaining adequate physical distance or choosing outdoor settings [45,47]. Gatherings should be avoided also during the use of changing rooms/common areas, and using showers should be deferred if possible [45]. Physical activity at school may be performed based on an epidemiological criterion (>50 new cases of confirmed cases within the district), as indicated by Simon et al. [48].

**School transports:** The use of public transport should be discouraged, advising the use of personal vehicles or promoting policies encouraging walking or cycling [46,47]. Behavioral rules to be adopted on board include disinfection after every trip and before picking up a new group of students, the use of masks and face shields for driver and staff, the maintaining of physical distancing, and following the respiratory etiquette and hand hygiene practice [45,46].

**School canteens:** Attendance of school canteens is discouraged by Bonell et al. [47]. Schools should ensure optimal hygiene while preparing lunches to prevent fomite-base transmission. If shared canteens remain open, then pupils should sit with a safe distance between them and different times of eating should be ensured for cohorts [45].

### 3.2. Management of Suspected and Confirmed Cases

#### 3.2.1. Official Documentation Released by Government Institutions

**Suspected case:** The first scenario that we examined consisted of a situation in which a student shows COVID-19-like symptoms at school (Table 5). Most documents reported that, in such cases, the COVID-19 contact person of the school, which is specifically designated to manage situations related to COVID-19, should be immediately called to handle the subsequent steps [26,27,34,36,43]. In all cases, parents/guardians should be called immediately to collect the student and, in the meantime, the student should be isolated. Most documents stated that a room or an area of the school should be identified and designated to be used as an isolation place [26,30,34,36,39,40,43]. In addition, some documents clarify that a designated isolation route should be followed to go to the isolation room [27,36]. Additionally, in Irish primary and special schools the isolation area does not have to be a room but it should be 2 m away from others [27]. The majority of documents point out that students showing symptoms should wear a mask [26,27,36,43], although according to France and Italy the mask should be worn only by students with symptoms with more than 6 years [23,34]. The student should stay in the presence of an adult, which should wear a mask too [23,27,30,34,36,40,43]. The adult should also keep a distance of at least 1 m [34] or 2 m [27,40]. In particular, some documents reported very specific recommendations for the equipment that the adult should wear depending on the situation. For instance, in the UK, in addition to a mask gloves should be worn if a contact with the student is necessary and eye protection should also be worn if the student is coughing, spitting or vomiting [40]. The Spanish document specifies that the adult can wear a surgical mask if the student has a mask too, otherwise the adult should wear a FFP2 mask [43]. In all cases, except for emergencies, the doctor or other designated healthcare/advice services should be called by parents/guardians at home as soon as possible. Portugal represents an exception since it is planned to call an ad hoc telephone line when the student is in the isolation room to have a telephone triage: if it is not a suspected case, the student should return in classroom or act accordingly to symptoms; if it is a suspected case, the student should isolate at home and contact primary health care or emergency department [36]. After the student leaves the school, the cleaning and disinfection of surfaces and areas must be carried out (only France and Luxembourg did not specify this issue). The Portuguese documentation adds that the products used by the student should be packed and placed in the waste after 24 h [36]. In the UK, all schools have a supply of home test kits to be offered in the exceptional circumstance that the school believes an individual may have barriers to accessing testing [39]. Parents/guardians should contact a healthcare provider to know if the student should be tested, such as General Practitioners (GPs) or family pediatricians [23,27,30,34] or in general primary care providers and local health authorities [36,43]. It must be noted that, in France, there is an online platform that parents/guardians can use [23], and in the UK tests can be booked online [39]. In any case, the student should self-isolate until a healthcare provider prescribes a test or decides that the student is not a suspected case. In some documents, it is clarified whether also other people should self-isolate while the student is waiting for the test result (or for an evaluation that concludes the student does not need a test). Specifically, in Luxembourg nobody else needs to self-isolate [25], in Ireland households contacts should be removed from school settings [30], in Portugal the Local Health Authority can decide to isolate close contacts [36], in the UK all household members should self-isolate [39], and in Spain siblings who attend the same or other schools should self-isolate [43]. Regarding the return to school (except the case when the student is tested positive), not all documents specify when the student can return if the test has not been prescribed or the student tested negative. The criteria to return are shown in Table 5.

In each country, the student should not go to school in case the symptoms appear outside school. The procedure to follow after the presentation of symptoms matches the procedure outlined above. Regarding staff members, in each country, they should immediately go home if symptoms appear at school (or do not go to school if symptoms appear outside school), self-isolate and call healthcare providers to receive instructions and get tested if COVID-19 is suspected. The procedure to follow is comparable to the procedure recommended for students. In some cases, it is specified that school workers have priority access to testing [34,39].

**Confirmed case:** In case the student is confirmed to be positive, the return-to-school criteria defined by the countries were mostly different, as reported in Table 6. For instance, the isolation goes from 7 days [23] to at least 10 days [39,43]. In all cases, students should return to school without symptoms, however only Italy and Portugal recommend that two tests should be negative before re-entry [34,36].

All the countries consider the quarantine of close contacts in school (staff and students), except Luxembourg, and report that the school should provide a list of contacts to health authorities, which perform risk assessment, epidemiological investigation, contact tracing, and guide the actions to be followed. When specified, the duration of quarantine can range from 7 days [23] to 10 [43] and 14 days [34,39]. Tests for close contacts are always required in France (staff and high school students) [23] and Luxembourg (entire class) [25]. In Luxembourg, no contact should be isolated and the entire class should be tested. Pending the results, they should follow more restrictive preventive measures: staff/students aged > 6 years must always wear a mask for 6 days from the last contact and until they test negative; staff/students should avoid school transports (or wear mask if not possible); students should be kept distant from students of other classrooms (e.g., no sports, breaks and lunchtime); staff/students are recommended to reduce social contacts [25]. In Italy, a special disinfection of involved school areas is specified [34]. In all countries, an entire school should not be closed for a single confirmed case. When specified [23,34,39], the actions to be followed in case a staff member tests positive are comparable to the actions implemented for a positive student.

Some documents clarify also the measures that must be taken if more than one case in one classroom is confirmed. In Luxembourg, if the transmission is identified outside school the base scenario remains the same and the involved classroom is quarantined and tested; if the transmission is within school, more restrictive measures should be implemented by a specific committee [24]. In some cases, it is specified that health authorities should be involved if there are many suspected absences within a classroom in order to plan a strategy [34,36,37,39]. For instance, among the possible strategies the Portuguese document lists: quarantine suspected and confirmed cases, the preventive quarantine of close contacts, and the closure of one or more classrooms/one or more areas of the school/entire school [36,37]. Interestingly, in the UK a mobile testing unit may be dispatched to test contacts [39]. Lastly, in Spain, the usual measures should be implemented if there are more than three cases in one classroom or cases in more classrooms without epidemiological links. However, if there is an epidemiological link or the transmission is not controlled additional measures should be performed from closures of entire years to the closure of the entire school for at least 10 days [43].

#### 3.2.2. Rapid Systematic Review

**Suspected case:** These documents commonly disagree on measures to be implemented if suspected and confirmed cases occur within schools. For instance, only the German consensus statement requires the presence of a COVID-19 contact person at school [48]. A designated and pre-identified isolation room is recommended by the Indian Academy of Pediatric guidelines [46], whereas a normal empty room is suitable for the isolation of symptomatic children in the Norwegian guide [45]. The latter is also the only document clarifying the equipment to be worn by children and staff in the case of symptoms, suggesting that pupils at school years 1–7 may wear a mask if they feel comfortable. Staff should always wear a mask if it is not possible to maintain 2 m of distance, particularly if the pupil does not wish to wear a mask [45]. Moreover, only the German document clarifies that students with symptoms should be promptly collected by parents/guardians and thereafter seek medical advice [48]. None of the documents state any recommendation for managing those who had been in contact with children/staff showing symptoms. If a pupil has developed symptoms, German experts and Norwegian guidelines recommend staying home until 24 h of the symptoms’ resolution [45,48]. This is also recommended in the algorithm proposed in the American paper on the condition that the children had not been exposed to a confirmed case. If this is the case, then 14 days of quarantine is required and, if symptoms develop, a COVID-19 PCR has to be performed [50]. Instead, the French Society of Pediatrics advises self-isolation for children developing symptoms until the resolution of symptoms, whereas in the case of symptoms lasting for >3 days, COVID-19 testing is required [49] (Table 7).

**Confirmed case:** Regarding children who test positive, all the articles agree on isolating students, but return to school is differently managed. In some cases, a medical certificate is required [46,48,50] and local health or government authorities should be informed [46,50]. The isolation period can vary from 7 days [49] to 14 days [46], whereas the American article proposes that 24 h of being afebrile and the improvement of symptoms might be sufficient for re-entry [50]. The contacts of confirmed cases should be quarantined, as proposed in Norwegian and American documents [45,50]. Two articles have proposed general recommendations for managing possible outbreaks within classrooms and schools. The German document suggests cluster isolation within the school facility in the event of an infection cluster, to be managed by a committee of the school in cooperation with the responsible public health department. This strategy should have priority over the closure of the entire institution [48]. The French Society of Pediatrics recommends class closure if three pupils test positive in the same class [49] (Table 8).

## 4. Discussion

The present paper mainly aimed to provide an overview of examples of guidelines from the WHO European Region for the 2020/2021 academic year reopening of primary and secondary schools, focusing on measures for the prevention and management of suspected and confirmed COVID-19 cases. A rapid systematic review of scientific literature was also conducted to find further suggestions.

Both considering the official documents and papers identified in scientific databases, there was a large consensus concerning preventive measures, such as decreasing interaction between students through staggering timetables and creating bubbles, promoting respiratory etiquette and hand hygiene, and intensifying the ventilation and cleaning of all areas. Additionally, considering recommendations about physical activity, school transport, and canteens, there were no outstanding differences. Some differences were outlined concerning physical distancing, however the distance ranged from 1 m to 2 m as a minimum and there was agreement in reorganizing classrooms and other areas to facilitate increased distancing. Similarly, masks were overall mandatory or highly recommended for adults and secondary school students (especially when physical distancing is difficult to achieve), while masks were usually not recommended for primary school students (or a risk-based approach was advised). One of the main differences we found was regarding students at high risk due to their medical conditions. Indeed, in some cases there was flexibility between attendance in presence or remote education, while in other cases all students were expected to physically return to school without exceptions. We argue that a one size fits all approach for high-risk children should not be the chosen approach [51,52]. Several considerations must be taken into account, such as the financial, technological, educational, emotional, and mental resources of families. Families can have concerns both for the health of their vulnerable children physically going to school and concerns for the effect of not attending school on education, social–emotional development, and mental health. Additionally, evaluating the specific disease is important in order to assess if the potential risk may not be worth the benefit for the family [51,52]. Last, as recommended by a team of American pediatric transplant diseases experts, open communication between families and schools should be encouraged to facilitate any useful information regarding child issues, such as transitions back to school, expectations for attendance, and performance during the school year [53].

Regarding the management of suspected and confirmed cases, the official documents mostly agreed. The existence of a specific COVID-19 contact person in the school and the location of the first call to have a telephone triage were among the main differences in the first step to be followed. In addition, the criteria to return to school for suspected and confirmed cases were slightly different among countries (Table 5 and Table 6). However, the isolation and testing of cases and closed contacts were common strategies, although with different timing. Luxembourg represents a special case; indeed, no quarantine for close contacts is planned, but only more restrictive preventive measures while waiting for the test results [25]. The papers identified through the rapid review of scientific databases provided fewer details; for instance, the COVID-19 contact person was mostly absent, as well as the pre-identification of an isolation room. Interestingly, also in this case the criteria to return to school were diversified, thus showing that a consensus is missing. Notably, when specified, both official documentations and scientific papers agreed that the entire school closure should be considered as the very last resort.

Overall, the official documentation taken into account in the present overview included the recommendations outlined in the WHO, United Nations Educational, Scientific and Cultural Organization (UNESCO), and United Nations International Children’s Emergency Fund (UNICEF) document published in September [54]. Indeed, in this document, the main prevention measures were physical distancing between individuals (at least 1 m in community- or cluster-transmission scenarios) and groups (e.g., staggering timetables and using cohorts); the use of masks (<5 years: no mask; 5–11 years: risk-based approach; >11 years: like adults); promoting hand hygiene and respiratory etiquette (e.g., providing hand hygiene stations and scheduling); adequate ventilation and the regular cleaning of the school environment daily [54]. Additionally, considering the screening and management of symptomatic students and staff, the policy of “staying at home if unwell” is definitely shared [54]. However, one of the major differences is represented by the isolation of contacts. Although the quarantine of contacts is a pillar both for the WHO and the specific countries (except Luxembourg [25]), the WHO, UNESCO, and UNICEF document recommends a 14-day self-isolation [54], while such a duration varied from 7 days [23] to 10 [43] and 14 days [34,39] in the considered documents.

The present work has some limitations that must be acknowledged. Above all, language represented one of the main limitations, as well as the limited sources of information used. Indeed, besides the government, Ministry of Health, and Ministry of Education websites that were searched, additional official documentation might be found in other official websites. In addition, we decided to consider only state-level guidelines, therefore further details and differences that can potentially be highlighted in regional or local guidelines are missing. Similarly, the rapid review of scientific literature cannot provide an immediate and prompt update of recommendations, as scientific publications require a certain amount of time to be peer-reviewed. Moreover, conducting a rapid systematic review may be itself a limitation, with the search being less extensive and comprehensive than a systematic review. Nevertheless, the present paper had the primary aim of providing an overview of the possible guidelines to be implemented for the 2020/2021 academic year reopening and, through a detailed comparison, allowed us to highlight both the commonly shared recommendations and the mainly different recommendations. It also outlined the broad heterogeneity of details that state-level guidelines provide.

## 5. Conclusions

In conclusion, the present paper showed that the recommendations and guidelines for reopening primary and secondary schools in the 2020/2021 academic year were mostly in agreement considering the measures of prevention and management of suspected and confirmed cases. However, among the differences that were found, this overview also showed that there was no strict consensus on the criteria for the return to school of students that tested positive and, therefore, we suggest that evidence on which criteria are more effective in limiting the transmission should be gathered. Additionally, it is worth noting that the flexibility between attendance at school and remote education for high-risk children varied across countries and papers. In our opinion, special attention should be paid to high-risk children; in particular, a one size fits all approach should not be the chosen approach. Lastly, the fact that school closure was commonly considered as the very last resort for COVID-19 control is extremely important in view of the widespread consequences that have been reported due to school closures in the past months [10,11,12,13].

Further studies are required to evaluate the impact of the different recommendations on controlling COVID-19 transmission in schools during this autumn term, and expert meetings may be useful in order to discuss available evidence. Moreover, we argue that the human factor should be also evaluated along with the effectiveness of the strategies per se. Indeed, suboptimal compliance to hygiene practice has been reported both at the school level (e.g., limited handwashing facilities) [55] and the student level (e.g., behavior of handwashing) [56], and many parents may want to keep their children home [57]. Additionally, young people have their own view on their role in the pandemic; they can be concerned about their own safety and the safety of others, and they need clear and understandable information rather than being treated as passive recipients [58].

## Figures and Tables

**Table 1:** PICOS strategy.

PICOS Strategy	
Population	Children attending primary and secondary school
Intervention	Prevention measures and/or management of suspected cases in school in the COVID19 pandemic
Comparison	None
Outcome	Not applicable
Studies	Guidelines, recommendations, protocols

**Table 2:** Information about the selected countries.

Country	First Lockdown Measures Implemented	School Closure	School Reopening for Academic Year 2020/2021
France [14,19,20]	17 March 2020	16 March 2020 * 6633 cases 148 deaths	1 September 2020 286,007 cases 30,661 deaths
Ireland [14,19,20]	27 March 2020	12 March 2020 70 cases 1 death	31 August 2020 28,811 cases 1772 deaths
Italy [14,19,20]	10 March 2020	10 March 2020 10,156 cases 633 deaths	14 September 2020 288,761 cases 35,624 deaths
Luxembourg [14,19,20]	18 March 2020	16 March 2020 * 68 cases 1 death	15 August 2020 6097 cases 123 deaths
Malta [14,19,20]	12 March 2020	13 March 2020 12 cases 0 deaths	28 September 2020 3006 cases 32 deaths
Portugal [14,19,20]	19 March 2020	16 March 2020 * 7374 cases 1 death	14 September 2020 68,208 cases 1871 deaths
Spain [14,19,20]	14 March 2020	16 March 2020 63,386 cases 354 deaths	7 September 2020 595,766 cases 30,585 deaths
United Kingdom [14,19,20]	24 March 2020	20 March 2020 * 3612 cases 186 deaths	1 September 2020 337,168 cases 41,504 deaths
San Marino [14,20,21]	9 March 2020	24 February 2020 0 cases 0 deaths	7 September 2020 716 cases 42 deaths

* Some schools were reopened before the end of the academic year. Dates of school reopening may slightly differ across the country according to local decisions.

**Table 3:** Preventive measures: official documentation released by government institutions.

Country	Minimum Physical Distance	Mask at School: Staff	Mask at School: Students
France [22]	Inside school: 1 m Outside school: not mandatory	Mandatory both indoor and outdoor	Primary school: not recommended Secondary school: mandatory both indoor and outdoor
Ireland [27,28,29]	Between students: 1 m between desks or single students Between staff: 2 m Between staff and students: 1 m, if possible 2 m	Mandatory if 2 m distancing not possible	Primary school: not recommended >13 years: not requested but not discourage wearing if distancing is difficult to maintain
Italy [31,32]	Between students: 1 m Between staff: 1 m Between staff and students: 2 m between teachers’ desk and students	Mandatory for any adult entering school	>6 years: mandatory in dynamic situations and if distancing not possible
Luxembourg [24]	Between staff: 2 m	Mandatory between adults if distancing not possible	Primary school: facultative inside classroom and during breaks Secondary school: mandatory during breaks; at the discretion of single school inside classroom
Malta [26]	Between students: 1.5 m in all directions whilst sitting Between staff: 2 m Between staff and students: 2 m	Required	>3 years: children should be advised to wear masks and/or visors in common areas; not necessary within their own classrooms or cluster >11 years: recommended use of masks and/or visors at all times, including in their classroom
Portugal [36,37]	Between students: 1 m Between staff and students: 1 m	Mandatory	>10 years: mandatory
Spain [41,42]	Between students: 1.5 m Between staff: 1.5 m Between staff and students: 1.5 m	Primary school: optional within group/bubble; mandatory outside those groups if distance less than 1.5 m Secondary school: mandatory if distance less than 1.5 m	>6 years: mandatory
UK [39]	Between students: support to maintain distance and not touch staff and peers Between staff: 2 m Between staff and students: 2 m	Not recommended universal use of face coverings. If distancing not possible, headteachers can decide to ask staff/visitors to wear face coverings in communal areas	Primary school: not necessary If distancing not possible, headteachers can decide to ask children in years 7 and above to wear face coverings in communal areas
San Marino [44]	Between staff and students: 1 m	Mandatory in common areas; mandatory inside classroom if distancing not possible	Primary school: facultative Secondary school: mandatory when entering and leaving school

**Table 4:** Preventive measures: rapid systematic review.

Author	Minimum Physical Distance	Mask at School: Staff	Mask at School: Students
Bonell et al. [47]	2 m between desks	N95 surgical masks (where supplies are short should be prioritized for clinical and social care staff)	In secondary schools
Cohen et al. [49]	NA	Mandatory at all times	In secondary schools. Less restrictive in classes where physical distancing is possible
Ghate et al. [46]	Always at least 1 m for all	Compulsory for all	Compulsory for all
Johansen et al. [45]	At least 1 m for all	Only when pupils/staff become ill and it is not possible to maintain a distance of 2 m	Only when pupils/staff become ill and it is not possible to maintain a distance of 2 m
Simon et al. [48]	1.5 m if high rates of transmission ^1^	Cloth face mask if distance rules cannot be observed	>10 years: recommended 6–10 years: recommended if high rates of transmission ^1^

Abbreviations: NA, Not Available ^1^ Defined as >50 new cases per 100.000 inhabitants of confirmed COVID-19 cases within the district in last 7 days.

**Table 5:** Management of a student with symptoms at school: official documentation released by government institutions.

Country	COVID Contact Person at School	Pre-Identified Isolation Room/Area	Student Equipment	Staff Equipment	First Call to a Doctor or Dedicated Service	Waiting for Results: Isolation of Other People	Return to School (If Not Tested Positive)
France [23]	NA	NA	If age > 6 years: mask	Mask	Home	NA	If not tested: Parents must certify they have consulted a doctor. Otherwise, after 7 days if symptoms disappeared.
Ireland [27,28,30]	Yes	Yes	Mask	Mask, at least 2 m	Home	Household members (removed from schools)	If tested negative: return when clinically well enough (all diarrhoea symptoms need to have been resolved for 48 h prior to return). Doctor’s certificate not required; only details as necessary for safe management are shared.
Italy [34]	Yes	Yes	If aged > 6 years: mask (in absence of mask: respiratory hygiene)	Mask, at least 1 m	Home	NA	If tested negative: stay home until symptoms disappearance. The doctor can decide to repeat the test after 2–3 days. Doctor’s certificate required.
Luxembourg [24,25]	NA	NA	NA	NA	Home	No	NA
Malta [26]	Yes	Yes	Mask	NA	Home	NA	Stay home until 24 h after symptoms resolve or as directed by public health authorities. Doctor’s certificate may be required.
Portugal [36,37]	Yes	Yes	Mask	Mask	School	Decision by Local Health Authority	NA
Spain [42,43]	Yes	Yes	Mask	Mask	Home	Siblings	If tested negative: return.
UK [39,40]	NA	Yes	NA	2 m distancing, if not possible: suitable PPE	Home	Household members	If tested negative: return when no more symptoms.

Abbreviations: NA, Not Available; PPE, Personal Protective Equipment.

**Table 6:** Management of a student confirmed positive: official documentation released by government institutions.

Country	Return to School	Main Strategies
France [23]	Student cannot return before the timing defined by the doctor (as soon as possible, 7 days after the test or onset of symptoms).	Staff/students of high school in the contact list must self-isolate and be tested 7 days after the last contact to return to school. Other students in the list: isolation for 7 days, test is not mandatory.
Ireland [27,28,30]	NA	Public health services discuss with the school any appropriate quarantine. Every facility will be unique. Close contacts: self-isolated, tested (at day 0 and 7) (no blanket policy to quarantine/test entire classes or years).
Italy [34]	Student returns if no symptoms and two negative tests at 24-h intervals.	Close contacts: 14 days of quarantine starting from the last contact. Prevention Department decides the most appropriate strategies for possible tests in students/staff.
Luxembourg [24,25]	NA	The entire class should be tested, staff included (no later than 6 days after the last contact). No isolation but more restrictive preventive measures until the results.
Malta [26]	NA	Students/staff that were contacts would need to go into quarantine.
Portugal [36,37]	Isolation until 3 consecutive days without fever and 1 negative rRT-PCR at least 14 days from the onset of symptoms (if no hospitalization) or 2 consecutive negative rRT-PCR (if hospitalization).	Local Health Authority can decide: contacts isolation and epidemiological investigation, closure of classroom/specific areas, environmental isolation.
Spain [42,43]	Isolation until 3 days after the disappearance of symptoms and a minimum of 10 days from the onset of symptoms.	Close contacts: 10 days quarantine from the last contact; recommended test after 10 days from the last contact; if the test is performed before the 10th day, the quarantine must be followed until the 10th day. The classroom will be closed for 10 days if the case belonged to a bubble. If the case did not belong to bubble: quarantine of close contacts only.
UK [39,40]	Isolation at least 10 days from the onset of symptoms; students return only if they do not have symptoms other than cough or loss of sense of smell/taste.	Close contacts should self-isolate for 14 days from last contact. If close contacts develop symptoms in the 14 days, they should get tested: -if negative, isolation for the remainder of the 14 days; -if positive, inform their setting and isolate for at least 10 days from the onset of their symptoms and their household should self-isolate for at least 14 days from when the symptomatic person first had symptoms.

Abbreviations: NA, Not Available.

**Table 7:** Management of a student with symptoms at school: rapid systematic review.

Author	COVID Contact Person at School	Pre-Identified Isolation Room/Area	Student Equipment	Staff Equipment	First Call to a Doctor or Dedicated Service	Waiting for Results: Isolation of Other People	Return to School (If Not Tested Positive)
Cohen et al. [49]	NA	NA	NA	NA	NA	NA	Stay home until symptoms resolve. If symptoms last for >3 days perform testing
Ghate et al. [46]	NA	Yes	NA	NA	NA	NA	NA
Johansen et al. [45]	NA	No	Age > 7: mask Age < 7: mask if pupil is comfortable	Mask if 2 m distance not possible	NA	NA	NA
Orscheln et al. [50]	NA	NA	NA	NA	NA	NA	If exposure to confirmed case, the student should be tested: if negative, return after 24 h without fever and symptoms improving If no exposure, evaluation by healthcare provider to get tested
Simon et al. [48]	Yes	NA	NA	NA	Home	NA	Stay home until 24 h symptoms resolve. Parents must confirm their child was free of symptoms for 24 h before being readmitted

Abbreviations: NA, Not Available.

**Table 8:** Management of a student confirmed positive: rapid systematic review.

Author	Return to School	Main Strategies
Cohen et al. [49]	After 7 days and possibly longer if symptoms persist. PCR monitoring is not necessary to return	Screening of entire class is only warranted if one teacher tested positive or if at least two children in the class are symptomatic and tested positive
Ghate et al. [46]	Notification to government authorities. Stay home for 14 days. Doctor’s certificate required	NA
Johansen et al. [45]	NA	NA
Orscheln et al. [50]	Return to school after 24 h afebrile and symptoms improving and approval of local health department. Doctor’s certificate required	Quarantine contacts of confirmed cases
Simon et al. [48]	Doctor’s certificate only required if child has been quarantined, a COVID-19 detection without symptoms or a close contact with a positive person. Institutions are not entitled to request a “negative test” as a condition for re-entry	Children living in the same household do not have to be necessarily tested, but remain in quarantine. This is decided by the public health department

Abbreviations: NA, Not Available.

## Supplementary Materials

The following are available online at https://www.mdpi.com/1660-4601/17/23/8839/s1. Supplementary material S1: Search strings.
