# Supplementary material for: Reopening Schools during the COVID-19 Pandemic: Overview and Rapid Systematic Review of Guidelines and Recommendations on Preventive Measures and the Management of Cases
Source: Int J Environ Res Public Health. 2020 Nov 27;17(23):8839. doi: 10.3390/ijerph17238839 (PMC7731329; doi:10.3390/ijerph17238839)
Supplement: Supplementary file 1 [file ijerph-17-08839-s001.pdf]

SUPPLEMENTARY MATERIAL: Search Strings

- **Pubmed**

  ("severe acute respiratory syndrome coronavirus 2" [Supplementary Concept] OR severe-acute-respiratory-syndrome-coronavirus-2[tiab] OR SARS-Coronavirus-2[tiab] OR sars-cov-2[tiab] OR nCoV[tiab] OR novel-coronavirus[tiab] OR novel-corona-virus[tiab] OR new-coronavirus[tiab] OR new-corona-virus[tiab] OR 2019nCoV[tiab] OR 2019-nCoV[tiab] OR coronavirus-disease-2019[tiab] OR corona-virus-disease-2019[tiab] OR coronavirus-disease-19[tiab] OR corona-virus-disease-19[tiab] OR covid*[Title/Abstract] OR "Coronavirus"[Title/Abstract] OR "SARS-CoV-2"[Title/Abstract] OR "COVID-19"[Supplementary Concept]) AND (education*[Title/Abstract] OR school*[Title/Abstract] OR student*[Title/Abstract] OR scholar*[Title/Abstract] OR "Schools"[Mesh] OR "Students"[Mesh]) AND (return*[Title/Abstract] OR re-open*[Title/Abstract] OR reopen*[Title/Abstract] OR "back to"[Title/Abstract] OR re-entry[Title/Abstract] OR reentry[Title/Abstract] OR re-enter[Title/Abstract] OR reenter[Title/Abstract] OR return*[Title/Abstract] OR ((going[Title/Abstract] OR coming[Title/Abstract] OR go[Title/Abstract] OR come[Title/Abstract]) AND (back[Title/Abstract])))

- **Scopus**

  ((TITLE-ABS-KEY ("Corona pandemi*")) OR (TITLE-ABS-KEY ("severe acute respiratory syndrome coronavirus 2")) OR (TITLE-ABS-KEY (sars-coronavirus-2)) OR (TITLE-ABS-KEY (sars-cov-2) OR (TITLE-ABS-KEY (ncov)) OR (TITLE-ABS-KEY (novel-coronavirus)) OR (TITLE-ABS-KEY (novel-corona-virus)) OR (TITLE-ABS-KEY (new-coronavirus)) OR (TITLE-ABS-KEY (new-corona-virus)) OR (TITLE-ABS-KEY (2019ncov)) OR (TITLE-ABS-KEY (2019-ncov)) OR (TITLE-ABS-KEY (coronavirus-disease-2019)) OR (TITLE-ABS-KEY (corona-virus-disease-2019)) OR (TITLE-ABS-KEY (coronavirus-disease-19)) OR TITLE-ABS-KEY (corona-virus-disease-

19)) OR (TITLE-ABS-KEY (covid*)) OR (TITLE-ABS-KEY ("Coronavirus")) OR (TITLE-ABS-KEY ("SARS-CoV-2")) OR (TITLE-ABS-KEY ("COVID-19"))) AND (((TITLE-ABS-KEY (education*)) OR (TITLE-ABS-KEY (school*)) OR (TITLE-ABS-KEY (student*)) OR (TITLE-ABS-KEY (scholar*)))) AND ((((TITLE-ABS-KEY (return*)) OR (TITLE-ABS-KEY (re-open*)) OR (TITLE-ABS-KEY (reopen*)) OR (TITLE-ABS-KEY ("back to")) OR (TITLE-ABS-KEY (re-entry)) OR (TITLE-ABS-KEY (reentry)) OR (TITLE-ABS-KEY (re-enter)) OR (TITLE-ABS-KEY (reenter)) OR (TITLE-ABS-KEY (return*)))) OR ((TITLE-ABS-KEY (going)) OR (TITLE-ABS-KEY (coming)) OR ((TITLE-ABS-KEY (go) OR (TITLE-ABS-KEY (come)) AND (TITLE-ABS-KEY (back))))))

- **Embase**

('covid-19':ti,ab,kw OR 'coronavirus':ti,ab,kw OR 'corona pandemi*':ti,ab,kw OR 'sars-cov-2':ti,ab,kw OR 'coronavirus disease 2019'/exp) AND ('education*':ti,ab,kw OR 'school*':ti,ab,kw OR 'student*':ti,ab,kw OR 'scholar*':ti,ab,kw OR 'student'/exp OR 'school'/exp) AND ('re-open*':ti,ab,kw OR 'reopen*':ti,ab,kw OR 'back to':ti,ab,kw OR 'back to school':ti,ab,kw OR 're-entry':ti,ab,kw OR 'reentry':ti,ab,kw OR 're-enter':ti,ab,kw OR 'reenter':ti,ab,kw OR 'return*':ti,ab,kw OR (('going':ti,ab,kw OR 'coming':ti,ab,kw OR 'go':ti,ab,kw OR 'come':ti,ab,kw) AND 'back':ti,ab,kw))
